# Ethical issues and best practice in clinically based genomic research: Exeter Stakeholders Meeting Report

**DOI:** 10.1136/medethics-2016-103530

**Published:** 2016-09-27

**Authors:** D Carrieri, C Bewshea, G Walker, T Ahmad, W Bowen, A Hall, S Kelly

**Affiliations:** 1 Egenis, Department of Sociology, Philosophy and Anthropology, University of Exeter, Exeter, UK; 2 IBD and Pharmacogenetics Research Group, Royal Devon and Exeter NHS Foundation Trust, Exeter, UK; 3 Health Research Authority, NHS, London, UK; 4 PHG Foundation, Cambridge, UK

**Keywords:** Clinical Ethics, Genethics, Patient perspective, Research Ethics, Regulation

## Abstract

Current guidelines on consenting individuals to participate in genomic research are diverse. This creates problems for participants and also for researchers, particularly for clinicians who provide both clinical care and research to their patients. A group of 14 stakeholders met on 7 October 2015 in Exeter to discuss the ethical issues and the best practice arising in clinically based genomic research, with particular emphasis on the issue of returning results to study participants/patients in light of research findings affecting research and clinical practices. The group was deliberately multidisciplinary to ensure that a diversity of views was represented. This report outlines the main ethical issues, areas of best practice and principles underlying ethical clinically based genomic research discussed during the meeting. The main point emerging from the discussion is that ethical principles, rather than being formulaic, should guide researchers/clinicians to identify who the main stakeholders are to consult with for a specific project and to incorporate their voices/views strategically throughout the lifecycle of each project. We believe that the mix of principles and practical guidelines outlined in this report can contribute to current debates on how to conduct ethical clinically based genomic research.

Current guidelines on consenting individuals to participate in genomic research are diverse.^1^ The Research Governance Framework for Health and Social Care 2004 provides guidance to protect the dignity, rights, safety and well-being of research participants and states that informed consent is at the heart of ethical research.^2^ There is also some guidance around consenting when using tissue banks pertaining to the Human Tissue Act 2004,^3^ and very specific guidance from the Joint Committee of Medical Ethics around consent in medical genetics.^4^

However, there does not seem to be any guidance available for researchers and certainly not for participants concerning consent for genomic research. This creates problems for participants, who may not have the confidence to give their consent, and also for researchers/clinicians who provide both clinical care and research to their patients. Current approaches to consent do not sufficiently take into account researchers' or participants' interests, and the specific issues that genomic research raises; for example, this research often blurs the boundaries with research and clinical care.

A group of 14 stakeholders met on 7 October 2015 in Exeter to discuss the ethical issues and the best practice arising in clinically based genomic research, with particular emphasis on the issue of returning results to study participants/patients in light of research findings affecting research and clinical practice. The group was deliberately multidisciplinary to cover diverse views and experiences. It included a general practitioner (GP), patient representatives, clinical genetic specialists, clinical research team members, molecular biologists, a lawyer, a genetic biobank manager, a representative of the Health Research Authority, members of the local 100 000 Genomes team, social scientists and ethicists. This meeting was organised as part of a collaboration between the Exeter IBD and Pharmacogenetics Research group and social scientist at the University of Exeter. This collaborative project titled ‘Consent and clinical trials in pharmacogenetics—facilitating the clinical implementation of modern genetic technologies for the treatment of patients with inflammatory bowel disease was funded by the ESRC Impact Cultivation Award.

We sought to identify both areas of best practice and the underlying ethical principles of such research. Our a priori agenda was to generate ethical guiding principles that were flexible rather than formulaic, in order not to deter good quality research and to enable it to be carried out ethically (ie, to respect the interests and rights of the stakeholders involved in the research process). We recognise that similar efforts are being carried out by other groups in the UK^5^ and other countries,^6^ indicating the importance and timeliness of issues involved, and the need for guidance, or at least clarity.

## Clinically based genomic research and ethical issues

We identified that clinically based genomic research carries particular characteristics^i^, from which ethical issues arise. Such research typically involves the potentially complicated relationships of clinician/researcher and patient/participant. The implications of these relationships need to be taken into account when research projects are designed.

Feeding back individual findings to research participants, who are also patients, may raise particular issues for clinicians, researchers and participants concerning ongoing and future treatment decisions. Participants'/patients' genetic data are potentially identifiable.

Genomic research has the potential to generate incidental findings (IFs), that is, findings that were not an intended objective of the study but were discovered as a consequence of the current technologies employed in this field. Such occurrences are rare although the consequences of imparting this information to study participants can be life-changing. It is difficult for clinicians and researchers to assess the validity and the clinical relevance of these results, to attribute the appropriate level of risk and to convey this information to participants in a meaningful way. However, research ethics committees expect clinicians and researchers to address the return of research findings to participants/patients in their research proposals (ie, to have a policy on returning summary or aggregated research results).

Significance and actionability criteria tend to be medically/treatment driven. Researchers/clinicians and patients/participants may have different views about the value of genetic information. For example, some participants may wish to know about *any* result generated by research—even if it is not clinically actionable—as it may have a life-related value for them (they may wish to have this knowledge for its own sake, for members of their family, etc).

Primary care physicians—GPs in the UK—are normally informed about their patients' participation in research. However, it is not clear what their roles and responsibilities are in relation to this information, and other information generated by genomic research. The custodianship of data and tissue donated to research are not always clear to research participants/patients and clinicians/researchers. There is no ownership of data and tissue, rather ownership of IP arising from analysis of the aforementioned.

## Examples of best practice

We identified the following examples of best practice. In the Nottingham Health Science Biobank (NHSB), experienced patients help research teams to take consent and are also consulted throughout research projects (eg, research teams seek their feedback in relation to information materials given to patients).^7^

The Exeter 10 000 project (EXTEND) biobank's model of governance uses a patient committee to approve researchers' access to blood samples and participant data on behalf of all participants, and contribute to the design of studies and information materials given to patients.^8^

Embedding NHS research and development departments in hospitals allows these departments to support research projects and research teams more effectively.

## Principles and practical guidance

In terms of principles and practical guidance underlying ethical clinically based genomic research, we identified the following.

### Trust

Trust is a key component, although its influence can be both positive and negative; established clinical relationships may both foster trust between the patient and clinician/researcher which facilitates research, but can also lead to a lack of clarity about whether the activity is part of clinical care or research, and to cutting corners in consent on both sides.

### Consultation

Consent is a process; it should be understood to be adaptable and consultative throughout the research project. Study design should be informed by consultations with patients, before patients are enrolled into the research. This would allow the identification of areas of concerns with regard to the intended procedures and ethical issues pertaining to the study. The key is genuine and timely consultation. Patient preferences regarding receiving primary and IFs will vary, between individuals and over time, and ‘one size fits all’ consent models and procedures should be avoided where possible. Approaches should take into account consultation and dialogue with stakeholders, including other healthcare professionals who may be involved.

Patients with relevant experience of a condition, research and/or treatment can be a valuable resource in the consent process. For example, they can be consulted by researchers who are setting up a new project to review participant-facing literature, or they can work alongside with research teams to promote interactive consenting processes.

Consultation should also include consulting the Health Research Authority (or similar organisations in other countries) to seek advice and guidance on acceptable practice.

Consent should not include promises regarding privacy and control over personal information in the event of data sharing, as they cannot be fulfilled. Patient/participant data cannot be deidentified (eg, pseudoanonymised and anonymised) in the clinical context, and data pathways should be transparent to participants. Researchers should ensure that their information governance is compliant with best current practices and legal guidance.

GPs are suitably placed to hold research and clinical information as their patient records are comprehensive, their IT systems are advanced and patient information contained therein follows the patient and is searchable (see eg, initiatives such as the Clinical Practice Research Datalink^9^). Consideration of GPs' role in terms of conveying and acting upon this information should be given.

Stakeholders, and how they are engaged, may vary depending on the specific projects. Therefore, ethical principles and guidelines (such as above) should guide researchers to identify who the main stakeholders are to consult with for a specific project and to incorporate their voices/views strategically throughout the lifecycle of the project. Ethical considerations should promote an open dialogue between the main stakeholders and a critical/genuine (as opposed to tokenistic) engagement of as many relevant stakeholders as possible.

### Training

Some stakeholders may need training to provide competencies for participating in such consultations.

### Technology

Ethical reasoning should not be tied too specifically to our current technology and capacity as these will soon become outdated.

The stakeholder meeting prompted the manager of the Exeter 10 000 project (EXTEND) biobank to consider how to responsibly prepare for the possibility of whole genome sequencing being conducted on the donors' tissue. The manager changed the information sheet and consent materials by adding more information and by making it more logistically easy for donors to withdraw consent. More generally, the meeting helped to establish links between different stakeholders and to lay the ground for future consultations.

It is our hope that this meeting report will inform other similar discussions about ethical issues in clinically based genomic research and we welcome responses.

## Footnotes

i We recognise that some of these characteristics are germane to genomic research, and they are also relevant to other types of research, for example, imaging studies.
